# A Synthetic Kinome Microarray Data Generator

**DOI:** 10.3390/microarrays4040432

**Published:** 2015-10-16

**Authors:** Farhad Maleki, Anthony Kusalik

**Affiliations:** Department of Computer Science, University of Saskatchewan, Saskatoon, SK S7N 5C9, Canada; E-Mail: kusalik@cs.usask.ca

**Keywords:** kinome array, synthetic data, normalization, heteroscedasticity of variance

## Abstract

Cellular pathways involve the phosphorylation and dephosphorylation of proteins. Peptide microarrays called kinome arrays facilitate the measurement of the phosphorylation activity of hundreds of proteins in a single experiment. Analyzing the data from kinome microarrays is a multi-step process. Typically, various techniques are possible for a particular step, and it is necessary to compare and evaluate them. Such evaluations require data for which correct analysis results are known. Unfortunately, such kinome data is not readily available in the community. Further, there are no established techniques for creating artificial kinome datasets with known results and with the same characteristics as real kinome datasets. In this paper, a methodology for generating synthetic kinome array data is proposed. The methodology relies on actual intensity measurements from kinome microarray experiments and preserves their subtle characteristics. The utility of the methodology is demonstrated by evaluating methods for eliminating heterogeneous variance in kinome microarray data. Phosphorylation intensities from kinome microarrays often exhibit such heterogeneous variance and its presence can negatively impact downstream statistical techniques that rely on homogeneity of variance. It is shown that using the output from the proposed synthetic data generator, it is possible to critically compare two variance stabilization methods.

## 1. Introduction

Phosphorylation and dephosphorylation are ubiquitous post-translational modifications of proteins. These processes, directed by protein kinases and phosphatases, cause conformational changes in the target protein, and lead to the regulation of protein function [1]. Since the functional activities of many proteins are interrelated, phosphorylation events may lead to complex signalling pathways that are involved in and coordinate those activities. To gain accurate knowledge about these complex signalling pathways and regulation mechanisms, it is essential to measure phosphorylation activities. Kinome microarray technology allows measurement of the phosphorylation activity of hundreds of proteins in a single experiment [2,3,4].

Researchers have used techniques from the area of transcriptional DNA microarrays to solve problems in the kinome microarray context. Since the chemistry involved in kinome microarray technologies differs from that of DNA microarrays, data analysis techniques appropriate for DNA microarrays may not be appropriate for kinome microarrays [5]. In addition, some characteristics of kinome arrays such as number of spots on an array and number of within-array replicates differ from those of DNA microarrays. These characteristics may affect downstream data analyses techniques. Therefore evaluating various algorithms and methodologies used by the DNA microarray community before applying them in kinome array data analysis is essential.

Synthetic kinome data generators would be a valuable tool to evaluate these algorithms and methodologies. Although there are some synthetic gene expression data generators [6,7], to the best of our knowledge there are no synthetic data generators designed for kinome microarrays. In this paper we propose such a simulator, and show its utility in quantitative evaluation of kinome array data analysis methods. Data produced by the simulator is used to evaluate Log2 and VSN for dealing with heterogeneity of variance, also called heteroscedasticity of variance, in kinome microarrays.

The available methods for simulating data for DNA microarrays may not be appropriate for simulating data with characteristics comparable to real kinome array data. For example, SIMAGE [8] was designed for dual-dye DNA microarrays and cannot be used for simulating data for kinome arrays, which are single channel. The method proposed by Dembélé [9] provides $log_{2}$ intensities or $log_{2}$ ratios, but in kinome array data simulation we are interested in raw data that provides both untransformed foreground and background intensity values, where background-corrected intensity values may be negative. This prevents the transformation of data generated by Dembélé’s method to kinome array data. The method proposed by Nykter *et al.* [10] is based on published error models for DNA microarrays. These error models have not been evaluated in the kinome array context and may not be applicable. In addition, the method requires the prudent selection of values for a large number (94) of parameters controlling the data generation. It is not clear what parameter values should be used for generating kinome array data, or how such values would be determined. DNA microarray data simulators are further described in Section 2.1.

Heterogeneity of variance is a common challenge confronting almost all types of microarray technologies. This includes kinome microarrays. It is problematic because the homogeneity of variance is an essential assumption for many statistical techniques including regression models and analysis of variance, and it may affect downstream microarray data analysis [11]. To eliminate or alleviate it in the context of microarray data analysis, variance-stabilizing methods are often used [12].

To the best of our knowledge, there are no variance stabilization methods designed for dealing with heterogeneity of variance in kinome microarray data. Therefore, available methods for dealing with this phenomenon in DNA microarrays have been used in the kinome array context [13,14]. Among these are the Log2 method [15] and variance-stabilizing normalization (VSN) [16]. These techniques are among the most widely used and studied methods for this purpose in DNA microarray community. They are described in more detail than that given here in Section 2.2.

The Log2 method transforms all positive values using a $log_{2}$ function and maps negative values to zero [12]. Although the method makes it easy to biologically interpret changes in measured intensity values, it suffers from several shortcomings. It ignores the measurement noise characteristics of the microarray data and does not utilize statistical information provided by within-array and between-array replicates. In addition, negative values, which are the result of background correction when the signal-to-noise ratio is low, cannot be handled by the $log_{2}$ function. Therefore, any negative values have to be mapped to zero, leading to information loss. Finally, Log2 inflates variance for low intensity measurements [12].

VSN is another widely used variance stabilization method in microarray data analysis [16]. VSN first brings different arrays to the same scale and then transforms the data in such a way that it shows an approximately constant variance across its entire range. This method, like the Log2 transformation, is capable of dealing with very high intensities. In addition, it acts much like a linear transformation for weak intensities. Therefore, it avoids the problem of variance inflation caused by the Log2 method for weakly expressed genes. The values between these two extreme situations are smoothly interpolated by VSN [13].

As mentioned, there are no variance stabilization methods designed for dealing with heterogeneity of variance in kinome microarray data, which are different from DNA microarray data from several aspects. These differences may affect the ability of variance stabilization methods to eliminate heterogeneity of variance in kinome array data. One of these differences is that kinome arrays do not have a statistically large number of within-array replicates like some DNA microarrays (e.g., Illumina arrays). Another is that kinome microarrays—unlike DNA microarrays, which usually contain thousands or tens of thousands of probes—contain only several hundred different peptides [14,17]. In fact, a kinome array is usually designed by selecting a set of functionally related peptides [18]. This functional dependency between phosphorylation activities of peptides on an array may make the overall distribution of kinome array measurements different from treatment to treatment, and this may affect the capability of variance-stabilizing methods to improve the data analysis.

Although variance-stabilization methods are used to eliminate or alleviate heterogeneous variance, the main goal of all preprocessing and normalization steps is to improve the result of downstream data analysis. It should be noted that a trivial way of stabilizing variance is to map data points to a constant value. Obviously, the problem with this trivial solution is that it destroys the information contained in the original data. This observation serves as a reminder that the choice of method for addressing heterogeneity of variance should be made by a cautious trade-off between the degree of variability and loss of information.

Considering all these factors, the natural questions are, which variance stabilization method should be used to deal with heterogeneity of variance in kinome microarray data? How do they affect the detection of differentially phosphorylated peptides? What is the effect of these transformations on fold-change? To answer these questions and many others, we require datasets for which correct analysis results are known. Unfortunately, such data is not readily available in the community. Further, there are no established techniques for creating artificial datasets with known results and with the same characteristics as real kinome datasets. In addition to data, we need criteria with which to evaluate the effects of various variance stabilization methods on kinome data analysis.

To provide kinome array datasets for which correct analysis results are known, we can generate synthetic kinome array datasets. Synthetic data are invaluable in evaluation and assessment of various systems, algorithms and scientific methodologies. Synthetic data sets make it possible to meet specific needs or certain conditions that may not be found in the original, real data. The main criticism of synthetic data is that it may be oversimplified or biased in a way that does not preserve characteristics of actual or original data. In this paper, we propose an algorithm to generate synthetic kinome array data that relies on actual intensity measurements from kinome microarray experiments to preserve subtle characteristics of the original kinome microarray data. One of these characteristics is within-array technical replicate variability. As will be shown, measurements for within-array technical replicates in the synthesized data have the same distribution as that of data from actual kinome arrays.

Furthermore, this paper suggests a quantitative framework to evaluate the effects of variance stabilization methods on detection of differentially phosphorylated peptides. In this regard, first, we propose a methodology for synthesizing artificial arrays, and then utilizing the artificial arrays with a priori knowledge of differentially phosphorylated peptides, we suggest a set of criteria for evaluating the variance stabilization methods. Finally, we use these methodologies to compare common variance stabilization methods.

The common approach to evaluating variance-stabilization methods is to compare the relation between mean and variance of data after applying each normalization method [12,19]. Instead, here we consider the effect of variance-stabilization methods on improving the results of downstream data analysis, which is the main goal of all normalization and preprocessing methods.

The rest of the paper is organized as follows. Section 2 provides additional detail on existing techniques for generating artificial DNA microarray data and why they are not suitable for kinome arrays. It goes on to discuss common methods to deal with heterogeneity of variance in microarray data. Section 3 presents the proposed method for synthesizing inter-array technical replicates and applying artificial phosphorylation such that the synthesized kinome array data reflects pre-determined phosphorylation patterns. Section 4 discusses criteria and a methodology for comparison of variance stabilization methods. Experimental design is described in Section 5, and Section 6 presents results of the analyses. Suggestions for future research are discussed in Section 7. Finally Section 8 ends the paper with a summary and conclusions.

Throughout the remainder of the document the terms “artificial array”, “synthetic array”, and “array” refer to kinome array data, rather than physical microarrays, unless otherwise stated.

green

## 2. Background

This section provides detail on existing tools for generating synthetic DNA microarray data, and on methods from the DNA microarray community that are used to address heteroscedasticity of variance.

### 2.1. DNA Microarray Data Simulators

There are several DNA microarray data simulators. Albers *et al.*, suggest SIMAGE, a model and web based software implementation for simulating dual-dye DNA microarray data [8]. Their proposed model requires the specification of up to 29 parameters including biological and technical parameters. They discuss that model parameters are strongly dependent on the experiment performed, and they may even vary in different experiments performed in the same laboratory. SIMAGE is designed for simulating dual-dye DNA microarray data and cannot be used for generating single channel microarray data. The authors state that due to specific properties of each type of DNA microarray, creating data simulators for other microarray platforms would be a useful and interesting direction for future research.

Dembélé proposed a model to simulate $log_{2}$ intensity data or $log_{2}$ ratio data for DNA microarrays [9]. As pointed out earlier, this is problematic for generating artificial kinome data where background-corrected values can be negative. In addition, model was constructed based on the assumption that intensities for each gene are uniformly distributed around its average. The noise component in the model is also normally distributed with a zero mean and a standard deviation, which is a parameter for the model. Therefore, generated microarray data from this method have a constant variance, which is not a realistic assumption for kinome microarray data, which suffer from heterogeneous variance; *i.e.*, there is a relation between mean and variance.

Nykter *et al.* [10] utilized several available error models to formulate biological and measurement variation in order to simulate microarray data with realistic characteristics. To represent the steps that may affect the quality of microarray data, they used noise, slide, hybridization, scanner, and error models. The models are controlled by multiple parameters, for a total of 94. It is not clear what parameter values should be used for generating kinome array data, or how such values would be determined. If nothing else, the task of determining values for such a large number of parameters discourages the method’s use.

### 2.2. Heteroscedasticity of Variance in Microarray Data Analysis

Heteroscedasticity of variance is a formidable challenge confronting almost all types of microarray technologies. Affymetrix GeneChip and Illumina Sentrix BeadChip arrays are advanced DNA microarray technologies. The former is widely studied and used [20], while the latter is relatively new [21]. The major difference between these two platforms is that the Illumina platform offers a larger number of within-array replicates that can be utilized for further analysis. Variance-stabilizing methods have been used to deal with heterogeneity of variance in Affymetrix arrays [16,19]. With the Illumina platform, the large number of within-array technical replicates has facilitated the use of additional methods [12].

VSN [16] and VST [12] are two variance-stabilizing methods used by the microarray community. These methods have been constructed based on a model for microarray gene expression measurement noise by Rocke and Durbin [22]. Their model is as follows:

(1) $$Y=\alpha +\mu e^{\gamma }+\epsilon$$

where *Y* is the measured intensity value, *α* is the average intensity of unexpressed genes, *μ* is the noise-free intensity value, *ϵ* is the additive error term, and *γ* is the multiplicative error term. The error terms are assumed to be normally distributed and independent random variables with zero means. Durbin *et al.* [19] utilized this model to introduce a transformation for variance stabilization in microarray data.

Huber *et al.* [16], employed the model suggested by Rocke and Durbin to design a variance-stabilizing method named VSN. VSN first brings different arrays to the same scale and then transforms the data in such a way that it shows an approximately constant variance across its entire range. This method, like the Log2 transformation, is capable of dealing with very high intensities. In addition, it acts much like a linear transformation for weak intensities. Therefore, it avoids the problem of variance inflation caused by the Log2 method for weakly expressed genes. The values between these two extreme situations are smoothly interpolated by VSN [16].

The VSN method was proposed prior to the advent of Illumina Sentrix BeadChip arrays. This platform offers a statistically large number of within-array replicates that can be utilized for variance stabilization. Lin *et al.* [12] utilized such replicates to estimate the parameters of the model suggested by Rocke and Durbin. The proposed method, named VST, uses the same transformation as VSN. The difference between these two methods is that they use different ways to estimate the model parameters [12].

Unlike Illumina microarrays, kinome microarrays do not provide a statistically large number of within-array replicates, yet the number of within-array replicates in these arrays is more than Affymetrix arrays. Previously, kinome microarrays provided 2 to 3 replicates for each probe [23]. This number is now about 9 replicates for each probe, which is three times more than in Affymetrix arrays. This difference between the numbers of within-array replicates may affect the ability of different variance stabilizing methods in eliminating heterogeneity of variance in kinome arrays.

## 3. Artificial Array Synthesis

The main purpose of kinome microarray experiments is to detect differentially phosphorylated peptides and therefore in kinome data analyses we are interested in preprocessing methods that minimize the error in classification of differentially phosphorylated and non-differentially phosphorylated peptides.

In order to compare variance-stabilizing methods with regard to peptide classification, we need kinome array datasets encoding a priori knowledge about which peptides are differentially phosphorylated. In this section we describe a methodology to synthesize such artificial arrays.

A main concern when synthesizing kinome arrays is to create datasets that reflect the characteristics of real kinome array measurements. One of these characteristics is the distribution of measurements for within-array replicates. On a kinome array, each peptide is represented by multiple spots, called within-array replicates. Although all within-array replicates for a peptide ideally should record the same level of phosphorylation, in practice the measurements vary.

Since the distribution of measurements for within-array replicates may significantly affect downstream data analyses, relying on any assumption about the distribution of these replicates when generating synthesized data may lead to over-simplification or misinterpretation of the resulting data.

### 3.1. Inter-Array Replicate Synthesis

In this subsection, we propose a method to synthesize inter-array technical replicates for a kinome array.

Suppose there exists a set of *n* kinome arrays each containing *m* different peptides (“probes”). In addition, suppose that there are *l* within-array replicates (“spots”) for each peptide. In the rest of the paper, the measurements for the $k^{th}$ within-array replicate of the $j^{th}$ peptide on the $i^{th}$ array are denoted by $r_{i,j,k}$. Variable $r_{i,j,k}$ contains all measurements corresponding to the intensity value of the aforementioned spot. More specifically, it contains foreground and background intensity values, denoted by $F(r_{i,j,k})$ and $B(r_{i,j,k})$, respectively. Also, the mean of a peptide is interpreted as the mean of the all background-corrected intensity values for all within-array replicates for that peptide.

We introduce the following notation to denote specific kinome arrays and the measurements from peptides on those arrays. Uppercase letters are used to denote a kinome array and its corresponding lowercase letter with a subscript is used to denote each peptide on the array. Following this notation a kinome array *A* is represented as follows:

$$\begin{array}{cc} A= & \left\{a_{j}|1\leq j\leq m\right\}\\ a_{j}= & \left\{a_{j,k}|1\leq k\leq l\right\} \end{array}$$

where *m* is the number of peptides on the array, *l* is the number of within-array technical replicates for each peptide, and $a_{j}$ is the set of all within-array technical replicates for the $j^{th}$ peptide on array *A*.

Our procedure to synthesize kinome arrays is described in technical detail in Algorithm 1 as the basis for rapid implementation. In the algorithm, $\left\{A_{1},\cdots ,A_{n}\right\}$ is a set of *n* peptide arrays, each containing *m* peptides and *l* within-array technical replicates for each peptide; *X* is a peptide array and is denoted by $X=\{x_{j}|1\leq j\leq m\}$; *T* is a threshold value for determining significant fold-change; *θ* is a percentage of noisy peptides; and *α* is a preferred statistical significance level. Each run of Algorithm 1 synthesizes an inter-array technical replicate of *X*; *i.e.*, an array with no differentially phosphorylated peptides when compared to *X*. That is, Algorithm 1 treats array *X* as a template, and produces inter-array replicates for it. The produced array is $Y=\{y_{j}|1\leq j\leq m\}$. In order to synthesize *q* inter-array technical replicates of *X*, the procedure is performed *q* times.

Algorithm 1 relies on real intensity measurements from kinome array experiments in order to preserve subtle characteristics of the original kinome microarray data. In addition, it allows the user to control the level of variation introduced using the fold-change threshold parameter, the percentage of noisy peptides, and the significance level parameters. In this regard, it creates a repository set *R* from existing kinome array data. This repository is sampled to synthesize an inter-array technical replicate *Y* for a given template *X*. We suggest using all available actual arrays to create the most comprehensive repository possible. The template *X* can be an array $A_{i}$ from that repository, though it need not be.
**Algorithm 1** inter-array Replicate Synthesis**Input:** $\left\{A_{1},\cdots ,A_{n}\right\}$, *X*, *T*, *θ*, *α***Output:** *Y*Create measurement repository set *R* from $\left\{A_{1},\cdots ,A_{n}\right\}$
$$\begin{array}{c} R=\left\{r_{i,j}|1\leq i\leq n,1\leq j\leq m\right\}\\ r_{i,j}=\left\{r_{i,j,k}|1\leq k\leq l\right\} \end{array}$$where $r_{i,j}$ is the set of all intra-array replicates of the *j*th peptide of $A_{i}$t←1**while** (t≤m) **do**
rand← a uniformly distributed random number between 0 and 1**if** rand≤θ **then**
$abc_{t}\leftarrow \frac{\sum_{k=1}^{l}F\left(x_{t,k}\right)-B\left(x_{t,k}\right)}{l}$**if** $abc_{t}\leq 0$ **then**
$v=\frac{abc_{t}}{T}$$w=abc_{t}\times T$**else**
$v=T\times abc_{t}$$w=\frac{abc_{t}}{T}$**end** **if****if** (there is a $r_{i,j}\in R$ where the mean of $r_{i,j}$ is not statistically bigger than *v* and not statistically less than *w*, considering a significance level of *α*) **then**
$y_{t}\leftarrow r_{i,j}$**else**
$y_{t}\leftarrow x_{t}$**end** **if****else**
**if** (there is a $r_{i,j}\in R$ where the mean of $r_{i,j}$ is not significantly different from the mean of $x_{t}$, considering a significance level of *α*) **then**
$y_{t}\leftarrow r_{i,j}$**else**
$y_{t}\leftarrow x_{t}$**end** **if****end** **if**t←t+1**end** **while****return** *Y*

The algorithm creates an inter-array technical replicate that should have no differentially phosphorylated peptide in comparison to a given template array. It uses fold-change to determine differential phosphorylation.

The obvious definition of fold change when the initial and final values are positive is

$$\mathrm{fold-change}=\frac{\mathrm{final}\mathrm{value}}{\mathrm{initial}\mathrm{value}}$$

We extend this definition to cover situations where both initial and final values are negative. A natural definition is as follows:

$$\mathrm{fold-change}=\frac{\mathrm{initial}\mathrm{value}}{\mathrm{final}\mathrm{value}}$$

Combining these expressions, in this paper we use the following generalized definition of fold change:

$$\mathrm{fold}-\mathrm{change}=\left\{\begin{array}{cc} \frac{\mathrm{final}\mathrm{value}}{\mathrm{initial}\mathrm{value}} & \mathrm{if}\mathrm{initial}\mathrm{value}>0;\&\mathrm{final}\mathrm{value}>0\\ \frac{\mathrm{initial}\mathrm{value}}{\mathrm{final}\mathrm{value}} & \mathrm{if}\mathrm{initial}\mathrm{value}<0;\&\mathrm{final}\mathrm{value}<0 \end{array}\right.$$

In Algorithm 1 any upward fold-change less than *T* and downward fold-change bigger than $\frac{1}{T}$ is considered as non-differential phosphorylation. These thresholds are captured by variables *v* and *w*, respectively. In addition, “significant difference” is defined as a difference that is statistically significant according to an independent one-sample t-test with a significance level of *α*.

Since *Y* should be an inter-array technical replicate of *X*, there must be no differentially phosphorylated peptides on *Y* in comparison to *X*. This is achieved in two different ways. For *θ* percent of the peptides, the algorithm introduces an amount of perturbation as follows. It calculates an average background-corrected intensity value, $abc_{t}$, for a peptide $x_{t}$. It then selects an arbitrary $r_{i,j}\in R$, where $r_{i,j}$ is not differentially phosphorylated in comparison to $x_{t}$ using a fold-change of *T* and confidence level of *α*. The algorithm adds $r_{i,j}$ as the *l* measurements for within-array replicates of the $t^{th}$ peptide in *Y* (*i.e.*, as $y_{t}$). Variables *w* and *v* are the bounds for determining whether the peptide represented by $r_{i,j}$ is not differentially phosphorylated. For the other 100-*θ* percent of peptides, the algorithm selects an arbitrary peptide $r_{i,j}\in R$, where $r_{i,j}$ is not significantly different from the mean of $x_{t}$, and assigns it to $y_{t}$. In both cases if such an $r_{i,j}\in R$ cannot be found, $y_{t}$ is set to the original $x_{t}$.

It should be noted that $r_{i,j}$ includes raw foreground and background intensity values.

### 3.2. Artificial Differential Phosphorylation

To generate synthesized arrays with known differentially phosphorylated peptides, we need a procedure to artificially phosphorylate or dephosphorylate a predetermined subset of peptides on an array. Here we propose a procedure to do this to array *Y*, which is an inter-array technical replicate of *X*, producing $Y^{\prime }$. The procedure is described in technical detail in Algorithm 2 as the basis for rapid implementation.
**Algorithm 2** Artificial Phosphorylation/Dephosphorylation **Input:**$A=\{A_{1},\cdots ,A_{n}\}$, *X*, *Y*, peptideIndex, phosphorylated, *T*, *α* **Output:**
$Y^{\prime }$ Create measurement repository set *R*
$$\begin{array}{c} R=\left\{r_{i,j}|1\leq i\leq n,1\leq j\leq m\right\}\\ r_{i,j}=\left\{p_{i,j,k}|1\leq k\leq l\right\} \end{array}$$ where $r_{i,j}$ is the set of all intra-array replicates of the $j^{th}$ peptide of $A_{i}$ $Y^{\prime }\leftarrow$ a copy of *Y* q←1 **while** (q≤ length(peptideIndex)) **do**  t←peptideIndex[q]  $abc_{t}\leftarrow \frac{\sum_{k=1}^{l}F\left(x_{t,k}\right)-B\left(x_{t,k}\right)}{l}$  **if**
$abc_{t}\leq 0$
**then**   $v=\frac{abc_{t}}{T}$   $w=abc_{t}\times T$  **else**   $v=T\times abc_{t}$   $w=\frac{abc_{t}}{T}$  **end**
**if**  **if** (phosphorylated[t] =1) **then**   **if** (there is a $r_{i,j}\in R$ where the mean of $r_{i,j}$ is statistically bigger than *v*, considering a significance level of *α*) **then**    $y_{t}^{\prime }\leftarrow r_{i,j}$   **else**    Report $t^{th}$ peptide as a non-differentially phosphorylated peptide   **end**
**if**  **else**   **if** (there is a $r_{i,j}\in R$ where the mean of $r_{i,j}$ is statistically less than *w*, considering a significance level of *α*) **then**    $y_{t}^{\prime }\leftarrow r_{i,j}$   **else**    Report $t^{th}$ peptide as a non-differentially phosphorylated peptide   **end**
**if**  **end**
**if**  q←q+1 **end**
**while** **return**
$Y^{\prime }$

In the Algorithm, $\left\{A_{1},\cdots ,A_{n}\right\}$ is a set of *n* kinome arrays, each containing *m* peptides and *l* within-array technical replicates for each peptide; *X* and *Y* are two inter-array technical replicates and are denoted by $X=\{x_{i}|1\leq i\leq m\}$ and $Y=\{y_{i}|1\leq i\leq m\}$, respectively; peptideIndex is a vector containing the indices of candidate peptides on *Y* to be (de)phosphorylated; phosphorylated is a binary vector with the same length as peptideIndex that shows the type of phosphorylation; *T* is a threshold value for significant fold-change; and finally *α* is a preferred significant level. In vector phosphorylated a value of 1 indicates phosphorylation while a value of 0 signifies dephosphorylation. It should be noted that $Y^{\prime }$ is not a technical replicate of *X* or of *Y*.

Algorithm 2 takes *X* and *Y* as input parameters and returns $Y^{\prime }$, which is a modified version of *Y*. Like Algorithm 1, Algorithm 2 creates a repository of kinome array measurements. For all *t* (1≤t≤m), if t∉peptideIndex , then $y_{t}^{\prime }$ must not be differentially phosphorylated and therefore $y_{t}^{\prime }=y_{t}$. For each t∈peptideIndex, to phosphorylate a peptide Algorithm 2 tries to select a $r_{i,j}\in R$ where $r_{i,j}$ has an upward fold-change bigger than *T* in comparison to $x_{t}$, and to dephosphorylate a peptide, tries to select a $r_{i,j}\in R$ where $r_{i,j}$ has a downward fold-change less than $\frac{1}{T}$ in comparison to $x_{t}$. The entire set of the *l* replicates, *i.e.*, $x_{t}$, is replaced by the set of *l* replicates of the chosen replacement peptide, $r_{i,j}$, which includes raw foreground and background intensity values.

It should be noted that in some cases it may not be possible to find an $r_{i,j}\in R$ that is differentially phosphorylated in comparison to $x_{t}$. This may happen because of a small and incomprehensive repository or because of an attempt to (de)phosphorylate a peptide that is highly (de)phosphorylated and further (de)phosphorylation is not possible. In this case, Algorithm 2 reports the peptide as not differentially phosphorylated and the value for $y_{t}^{\prime }$ remains unchanged.

Real kinome data tends to have approximately 10% to 15% of probes differentially phosphorylated [13,24,25]. Thus users should not specify a parameter peptideIndex with cardinality that is more than about 15% of the number of probes on template array *X*.

## 4. Criteria and Methodology for Evaluation of Performance Measures

As mentioned before, the main purpose of kinome array experiments is to detect differentially phosphorylated peptides. Variance stabilization is a preprocessing step to increase accuracy of various downstream analyses to detect such peptides. Therefore, we suggest and use a set of quantitative performance measures to evaluate the effect of variance stabilization methods on the performance of peptide classification.

### 4.1. Criteria for Evaluation of Performance Measures

In this paper, we use sensitivity, specificity, precision, and accuracy as performance measures for peptide classification [26]. We interpret the word “positive” as “differentially phosphorylated” and the term “negative” as “non-differentially phosphorylated”. In addition, we use the following notations:

- ∥∥ operator denotes the size of a set.
- TP (True Positives): the set of all differentially phosphorylated peptides predicted as differentially phosphorylated.
- FN (False Negatives): the set of all differentially phosphorylated peptides predicted as non-differentially phosphorylated.
- TN (True Negatives): the set of all non-differentially phosphorylated peptides predicted as non-differentially phosphorylated.
- FP (False Positives): the set of all non-differentially phosphorylated peptides predicted as differentially phosphorylated.

The specificity criterion shows the proportion of all true negatives classified correctly, and is defined as follows:

(2) $$\mathrm{Specificity}=\frac{∥\mathrm{TN}∥}{∥\mathrm{TN}∥+∥\mathrm{FP}∥}$$

The sensitivity score, which is also referred to as recall, shows the proportion of all positives classified correctly, and is defined as follows:

(3) $$\mathrm{Sensitivity}=\frac{∥\mathrm{TP}∥}{∥\mathrm{TP}∥+∥\mathrm{FN}∥}$$

The precision criterion shows the proportion of all true positive samples against all the positive results, and is defined as follows:

(4) $$\mathrm{Precision}=\frac{∥\mathrm{TP}∥}{∥\mathrm{TP}∥+∥\mathrm{FP}∥}$$

Accuracy is the proportion of all samples classified correctly, and is defined as follows:

(5) $$\mathrm{Accuracy}=\frac{∥\mathrm{TP}∥+∥\mathrm{TN}∥}{∥\mathrm{TP}∥+∥\mathrm{TN}∥+∥\mathrm{FP}∥+∥\mathrm{FN}∥}$$

### 4.2. Evaluation of Performance Measures

In order to compare the effect of variance stabilization methods on specificity, sensitivity, precision, and accuracy, we use the following procedure:

**Procedure:**  **Performance evaluation**
**Input:**  $\left\{A_{1},\cdots ,A_{n}\right\}$ , a set of *n* actual kinome arrays
  $n_{d}$, the maximum number of differentially phosphorylated peptides on each pair of arrays
  *T*, the threshold value for significant fold-change
  *θ*, percentage of noisy peptides
  *α*, a significance level
  $n^{\prime }$, number of synthesized arrays ($n^{\prime }\leq n$)
**Output:**  Calculated value of specificity, sensitivity, accuracy, and precision for each pair of inter-array technical replicates, and for each normalization method
**Step 1:**  For each *q*, where $1\leq q\leq n^{\prime }$, do Step 2 through Step 8:
**Step 2:**  Using Algorithm 1, create an inter-array technical replicate $Y_{q}$, where $Y_{q}$ is an inter-array technical replicate for $A_{q}$, considering $\left\{A_{1},\cdots ,A_{n}\right\}$, *T*, *θ* and *α*.
**Step 3:**  Phosphorylate a random subset of peptides on $Y_{q}$ using Algorithm 2, considering $\left\{A_{1},\cdots ,A_{n}\right\}$, $A_{q}$, *T*, and *α*; exclude peptides that cannot be differentially phosphorylated by Algorithm 2 from the random subset and record the differentially phosphorylated peptides regardless of their fold-change direction in a set $P_{q}$; name the output as $Y_{q}^{\prime }$.
**Step 4:**  For each normalization method do steps 5 to 8.
**Step 5:**  Normalize the pair $\left(A_{q},Y_{q}^{\prime }\right)$, and denote the normalized array pair $\left(A_{q}^{*},Y_{q}^{*}\right)$.
**Step 6:**  For the pair $\left(A_{q}^{*},Y_{q}^{*}\right)$ detect the phosphorylated peptides and save them in a set $F_{q}$.
**Step 7:**  Calculate true positive (*TP*${}_{q}$), false positive (*FP*${}_{q}$), true negative (*TN*${}_{q}$), and false negative (*FN*${}_{q}$) sets as follows:
  $$\begin{array}{cc} {\mathrm{TP}}_{q} & =P_{q}\cap F_{q}\\ {\mathrm{FP}}_{q} & =\left(N_{q}-P_{q}\right)\cap F_{q}\\ {\mathrm{TN}}_{q} & =\left(N_{q}-P_{q}\right)\cap \left(N_{q}-F_{q}\right)\\ {\mathrm{FN}}_{q} & =\left(P_{q}\right)\cap \left(N_{q}-F_{q}\right) \end{array}$$
  where $N_{q}$ is the set of all peptides on array $Y_{q}$.
**Step 8:**  Using ${\mathrm{TP}}_{q}$, ${\mathrm{FP}}_{q}$, ${\mathrm{TN}}_{q}$, and ${\mathrm{FN}}_{q}$, calculate specificity, sensitivity, accuracy, and precision.

## 5. Experimental Section

In order to illustrate the use of Algorithms 1 and 2 for comparing variance stabilization methods, we performed an experiment to evaluate the performance of two variance stabilization methods used in the context of kinome arrays. The two methods were Log2 and VSN [16]. This section describes the data and methodology used in that experiment.

The first step in the experiment was to obtain a collection of kinome microarray data. For this, a dataset from a published kinome array experiment for investigation of the existence of species- and individual-specific kinotypes in human and pig was used [27]. There were six biological replicates for each species, *i.e.*, twelve individuals in total. The study was run for four consecutive weeks leading to 48 arrays. Each array contained 297 peptides and 9 within-replicates for each peptide. More information about the design, construction and application of these arrays is given elsewhere [18,27].

In our experiment we used $n_{d}=30$, $n^{\prime }=48$, n=48, T=2, θ=0.05, and α=0.05 as the parameter values, unless otherwise stated. Furthermore, peptideIndex was set to represent a random subset of $n_{d}$ peptides, and phosphorylated was set to be a random binary vector of the same length as peptideIndex, *i.e.*, $n_{d}$.

Following the performance evaluation procedure in Section 4.2, for each actual kinome array $A_{q}$, Algorithm 1 was used to create an inter-array replicate $Y_{q}$. Then Algorithm 2 was used to differentially phosphorylate a subset of at most $n_{d}$ peptides on $Y_{q}$, resulting in creation of $Y_{q}^{\prime }$. Following this, using a variance stabilization method $A_{q}$ and $Y_{q}^{\prime }$ were normalized, leading to $A_{q}^{*}$ and $Y_{q}^{*}$. Then a paired t-test with a significant level of 0.05 was used to detect the differentially phosphorylated peptides on $Y_{q}^{*}$ in comparison to $A_{q}^{*}$. Finally, using the list of differentially phosphorylated peptides, $P_{q}$, the values of sensitivity, specificity, precision, and accuracy were calculated.

After calculation of all the criteria for all variance stabilization methods, we used a Levene’s test with significance levels of 0.05 to assess the equality of variances. Considering the result of Levene’s test a paired t-test with equal or unequal variance was employed to compare the effect of various variance stabilization methods on specificity, sensitivity, accuracy, and precision.

A Shapiro-Wilk test was used to assess the null hypothesis that the average background-corrected intensity values in kinome array data are normally distributed. In order to test the null hypothesis that an original kinome array and its synthesized differentially phosphorylated array generated by Algorithms 1 and 2 have the same distribution, we used two-sample Kolmogorov-Smirnov test. A significant level of 0.05 was used in both tests.

Algorithms 1 and 2 were implemented in the python programming language, while the data analysis was implemented in R [28]. Built-in R functions were used for the Shapiro-Wilk and two-sample Kolmogorov-Smirnov tests. The R package called “lumi” was used for VSN normalization [16]. The implementation of Levene’s test was provided by the “leveneTest” function in an R package called “car” [29]. The built-in R function “*t*-test” was used to compare the means of each criterion for both variance-stabilization methods. It should be noted that the “var.equal” parameter of “*t*-test” was set according to the result of Levene’s test to indicate whether to assume equality of variance in the “*t*-test”.

## 6. Results

The *p*-values for all 48 actual kinome arrays were less than the significance level of 0.05 (see Table S1 in Supplementary Materials). Therefore, the null hypothesis that the average background-corrected intensity values of actual kinome arrays are normally distributed is rejected. This further emphasizes that relying on normal distribution to simulate kinome array data may lead to unrealistic results.

**Figure 1:**
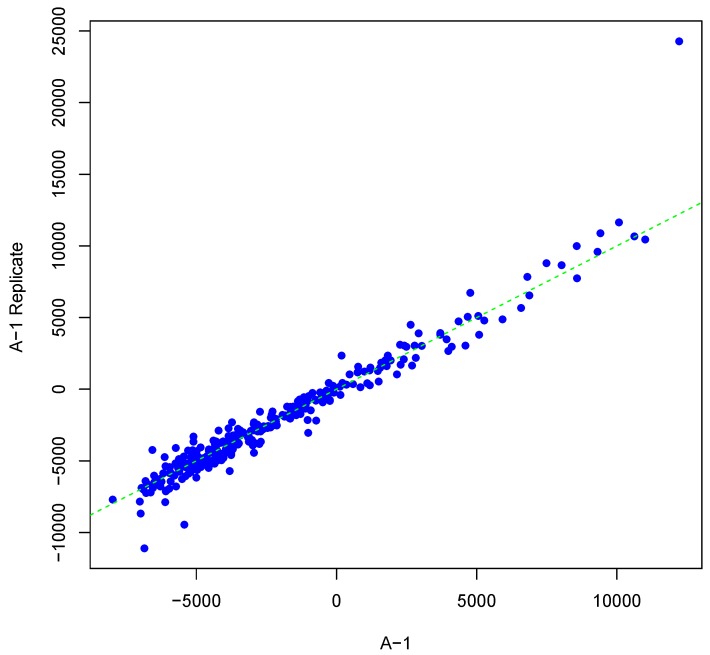
Scatter plot of background-corrected intensity values for an array and its synthesized inter-array replicate with *T* = 2 and *θ* = 0.05.

**Figure 2:**
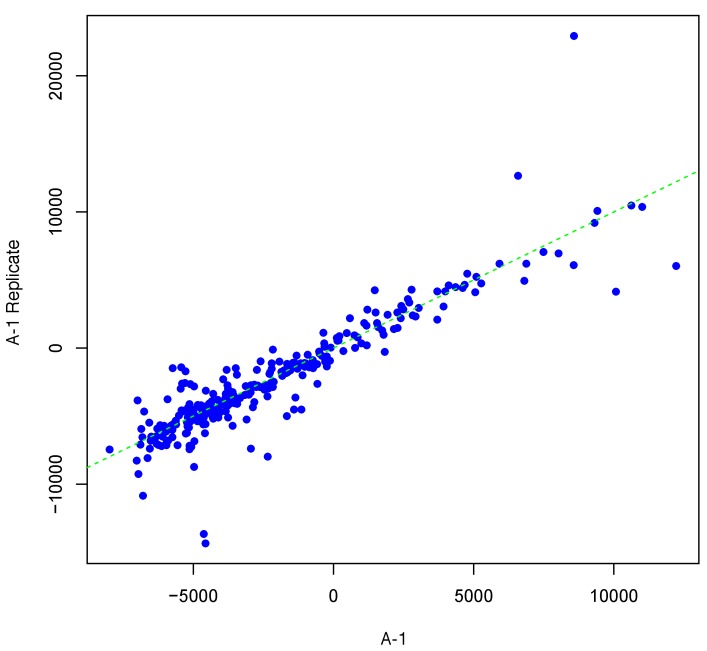
Scatter plot of background-corrected intensity values for an array and its synthesized inter-array replicate with *T* = 3 and *θ* = 0.10.

**Figure 3:**
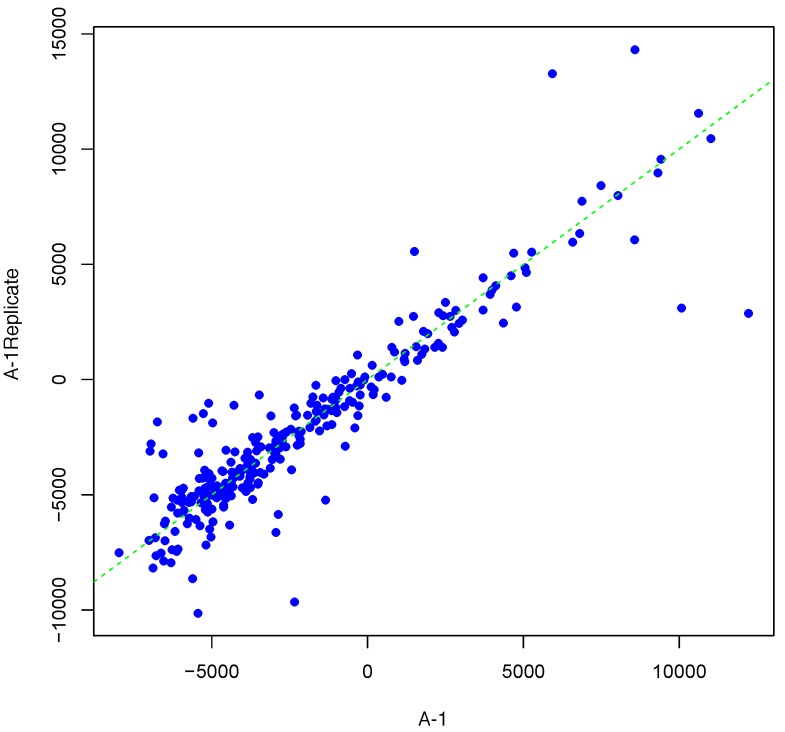
Scatter plot of background-corrected intensity values for an array and its synthesized inter-array replicate with *T* = 4 and *θ* = 0.15.

Figure 1 shows an example result of applying Algorithm 1 for generating an inter-array replicate to an actual kinome array, “A-1”. In absence of noise, measurements for each peptide on inter-array replicates would be the same, and all points would lay on the identity line. However, this does not happen in the real world due to many sources of variability. Algorithm 1 allows the user to control the level of variability in generation of a synthesized kinome array using the fold-change threshold, percentage of noisy peptides, and significance level parameters (*T*, *θ*, and *α*, respectively). The result is shown in Figure 1 by points that deviate from the diagonal y=x. Figure 2 and Figure 3 show inter-array replicates for the same starting array with *T* = 3, *θ* = 0.10, and *T* = 4, *θ* = 0.15, respectively. In all three plots, the horizontal axis corresponds to the actual array, the vertical axis corresponds to the synthesized inter-array replicate, and each point depicts the average background-corrected intensity values for a peptide.

Scatter plots of original versus replicate array pairs for three template arrays other than “A-1” in the input dataset are shown in Figures S1–S3. They visually demonstrate that the algorithm does not create replicates using a repeating pattern and that generated replicates are reminiscent of actual inter-array replicates.

**Figure 4:**
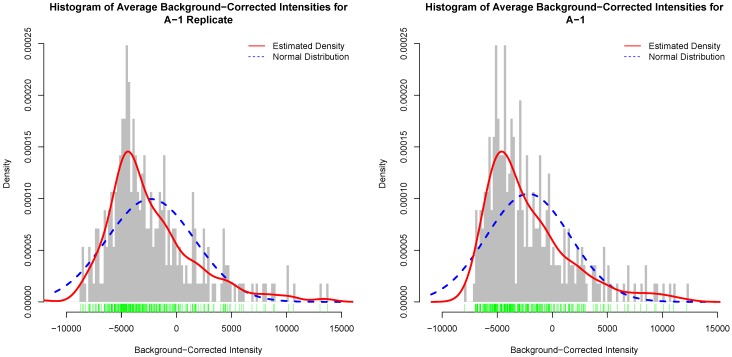
Histogram of background-corrected intensity values for an actual kinome array (**left**); and its inter-array technical replicate (**right**). The green bars show a one-dimensional plot of background-corrected intensity values. The red curve is the estimated distribution of the values.

Figure 4 depicts histograms of average background-corrected intensity values for an actual kinome array and its inter-array technical replicate. In this figure, the red curve is the estimated distribution of the average background-corrected intensity values [30]. Similar figures for the other template arrays and their replicates are given as Figures S4–S6. It is clear that the histograms for the actual array and its inter-array technical replicate are not unrealistically the same, and that they generally follow the same distribution as depicted by the estimated distribution of the data.

**Figure 5:**
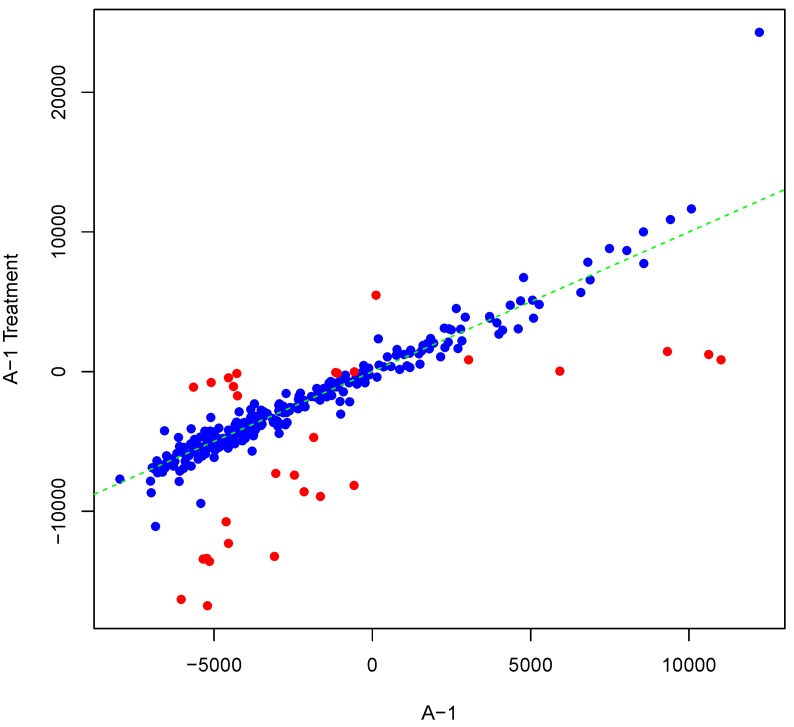
Scatter plot of background-corrected intensity values for an array and a phosphorylated version of its synthesized inter-array replicate when T=2. Differentially-phosphorylated peptides are depicted in red.

Figure 5 shows an example result of applying Algorithm 2 for differentially phosphorylating a set of peptides on the array “A-1”. The replicate, parameter *Y*, was as shown in Figure 1. Differentially phosphorylated peptides in Figure 5 are depicted in red. Again background-correct intensity values are plotted. It should be noted that although we set the number of candidate peptides for phosphorylation to be 30, *i.e.*, the length of phosphorylated vector in Algorithm 2, the number of differentially phosphorylated peptides is less than or equal to 30. This can happen because of an attempt to (de)phosphorylate a peptide that is highly (de)phosphorylated. This can lead to fewer differentially phosphorylated peptides than specified by $n_{d}$. Scatter plots of original versus artificially phosphorylated replicate array pairs for three template arrays other than “A-1” in the input dataset are shown in Figures S7–S9 in Supplementary Materials.

The null hypothesis for two-sample Kolmogorov-Smirnov test is that inter-array technical replicates produced by Algorithm 1 and the phosphorylated arrays produced by Algorithm 2 have the same distribution as the original (template) arrays. The *p*-values reported by the tests were greater than the significance level (0.05) in 46 of 48 cases for inter-array technical replicates, and in 41 of 48 cases for synthesized differentially phosphorylated arrays (see Tables S1–S3). Thus, in general the null hypothesis cannot be rejected.

**Figure 6:**
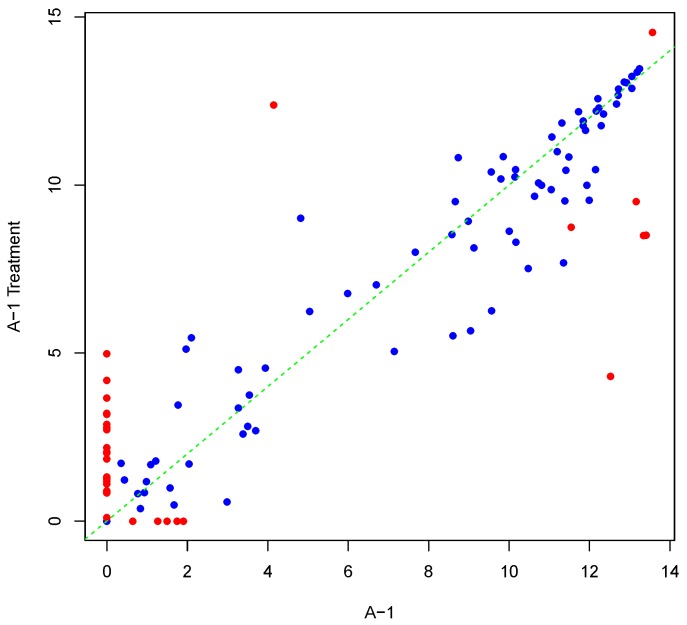
Scatter plot of background-corrected intensity values for an actual array and its generated, differentially-phosphorylated replicate, after Log2 normalization. Seeded differentially-phosphorylated peptides are depicted in red.

**Figure 7:**
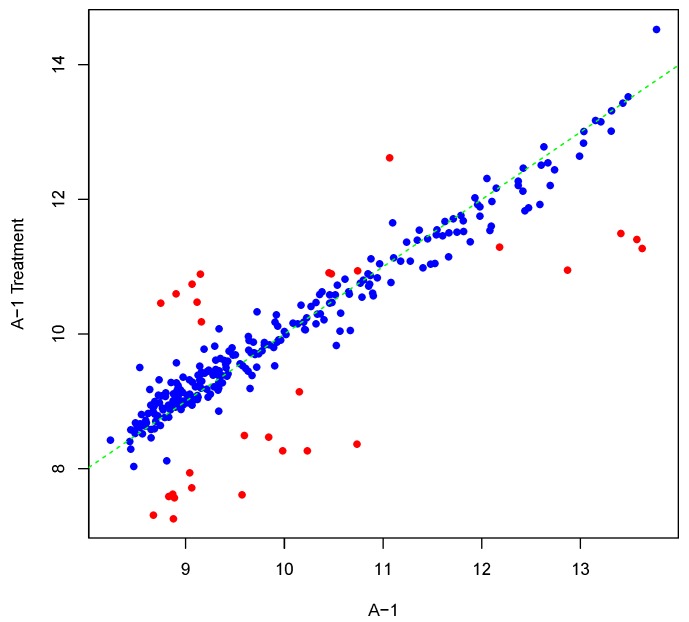
Scatter plot of background-corrected intensity values for an actual array and its generated, differentially-phosphorylated replicate, after VSN normalization. Seeded differentially-phosphorylated peptides are depicted in red.

Figure 6 and Figure 7 illustrate the effects of Log2 and VSN normalization, respectively, for the actual array and generated, differentially-phosphorylated replicate shown in Figure 5. In the scatter plots, the horizontal axis shows the actual array, while the vertical axis corresponds to the generated, differentially-phosphorylated replicate. The values on both axes were subjected to the transformation shown. In both figures the true differentially-phosphorylated peptides (set $P_{q}$ of Step 3 in Section 4.2) are coloured in red to differentiate them from other peptides. Comparing Figure 6 and Figure 7 to Figure 5, it is obvious that Log2 destroys the information content for nonpositive average intensity values, while VSN preserves almost the same pattern as the raw array. However, VSN does not maintain fold-change values.

In order to examine the effect of Log2 and VSN transformations on the detection rate of differentially phosphorylated peptides, we applied the performance evaluation procedure (Section 4.2) to generate 48 pairs of arrays, and for each performance measure, we conducted a Levene’s test (Table 1) and a paired *T*-test (Table 2) to determine whether there is a significant difference between means of that performance measure for Log2 and VSN. For all cases where the *p*-value in Levene’s test was less than the significance level (α=0.05), the hypotheses that the population variances are equal is rejected. Therefore, for all measures paired *t*-tests assuming unequal variances were performed. Table 2 illustrates the t-statistics and *p*-values from the paired *t*-tests. Moreover, it depicts the average value of each performance measure for the Log2 and VSN methods. In all cases the degrees of freedom for the *t*-statistic was 47. These results indicate that the accuracy, sensitivity, and precision performance measures were significantly higher for VSN than for Log2 transformation. This result is in accordance with other studies in transcriptional DNA microarrays that indicate superiority of VSN over Log2 [19,31].

**Table 1:** Levene’s test for equality of variances.

Performance Measure	*F*-Value	*p*-Value
Specificity	6.9639	0.0097360
Sensitivity	24.327	0.0000035
Accuracy	6.3493	0.0134294
Precision	9.3306	0.0029321

**Table 2:** Paired *t*-test for comparison of the difference in performance measures between Log2 and VSN.

Performance Measure	*t*	Log2 Mean	VSN Mean	*p*-value
Specificity	+2.7739	0.9026	0.8809	$9.960\times 10^{-1}$
Sensitivity	–31.529	0.3774	0.9601	$1.432\times 10^{-33}$
Accuracy	–5.7156	0.8520	0.8888	$3.617\times 10^{-7}$
Precision	–10.557	0.3096	0.5008	$2.703\times 10^{-14}$

## 7. Discussion

This paper suggests a quantitative framework to evaluate the effects of variance stabilization methods on detection of differentially phosphorylated peptides. This framework is not limited to variance stabilization methods; any preprocessing or normalization method can be evaluated by considering its effect on peptide classification.

The proposed kinome data generator simulates kinome microarray data that consists of foreground and background intensity value pairs. However, the proposed methodology can be used in situations where only foreground values are available. To simulated such a data, one should assume availability of backgrounds and assign zero to all background intensities.

In this paper we used *t*-test as a tool for detection of differentially phosphorylated peptides. Other detection methods are possible. Hence, a modified version of performance evaluation procedure to compare various methods for detecting differentially phosphorylated peptides is a suggestion for further research.

The visual representation of the VSN transformed arrays reveal that the VSN does not maintain fold-change values. Study of the effect of VSN transformation on various seeded fold-change values is suggested for future research. In addition, for the normalization technique, devising a method to rapidly find a fold-change in transformed data that is equivalent to a given fold-change in untransformed data would also be useful.

## 8. Conclusions

In this paper, we proposed a synthetic kinome array data generator to synthesize datasets for which correct analysis results are known. The proposed synthetic data generator allows the user to control the level of noise in generation of a synthesized kinome array using the fold-change threshold parameter and the significance level parameter. The proposed method also relies on actual intensity measurements from kinome microarray experiments to preserve subtle characteristics of the original kinome microarray data. Histograms and statistical tests of actual arrays and synthesized arrays indicate that the synthesized arrays follow the same distribution as that of actual kinome arrays. The utility of the algorithm was demonstrated by evaluating Log2 and VSN normalization methods.

In order to compare these methods, we considered their effects on improving the results of downstream data analysis, which is the main goal of all normalization and preprocessing methods. More specifically, since the main purpose of kinome microarray experiments is to classify differentially and non-differentially phosphorylated peptides, we used sensitivity, specificity, precision, and accuracy as performance measures for peptide classification. The statistical data analysis indicated the superiority of VSN over Log2 method for the accuracy, sensitivity, and precision performance measures. Although we used a quantitative approach for comparing variance stabilization methods, this result is in accordance with other research in the transcriptional DNA community.

## Supplementary Files

Supplementary File 1
