# Supplementary material for: A Synthetic Kinome Microarray Data Generator
Source: Microarrays (Basel). 2015 Oct 16;4(4):432–53. doi: 10.3390/microarrays4040432 (PMC4996406; doi:10.3390/microarrays4040432)
Supplement: Supplementary File 1 [file microarrays-04-00432-s001.pdf]

Supplementary Information

Table S1. Table of calculated test statistics and *p*-values from the Shapiro-Wilk test applied to each of the 48 initial arrays in the dataset used for the experiment.

Array Number	Statistic	*p*-Value
1	0.89	0.00
2	0.80	0.00
3	0.77	0.00
4	0.81	0.00
5	0.96	0.00
6	0.92	0.00
7	0.92	0.00
8	0.94	0.00
9	0.80	0.00
10	0.94	0.00
11	0.94	0.00
12	0.94	0.00
13	0.78	0.00
14	0.95	0.00
15	0.90	0.00
16	0.89	0.00
17	0.93	0.00
18	0.91	0.00
19	0.95	0.00
20	0.83	0.00
21	0.83	0.00
22	0.91	0.00
23	0.89	0.00
24	0.85	0.00
25	0.84	0.00
26	0.89	0.00
27	0.85	0.00
28	0.86	0.00
29	0.87	0.00
30	0.83	0.00
31	0.82	0.00
32	0.86	0.00
33	0.86	0.00
34	0.82	0.00
35	0.79	0.00
36	0.80	0.00
37	0.86	0.00
38	0.89	0.00
39	0.94	0.00
40	0.83	0.00
41	0.85	0.00
42	0.90	0.00
43	0.88	0.00

Table S1. *Cont.*

Array Number	Statistic	*p*-Value
44	0.91	0.00
45	0.91	0.00
46	0.86	0.00
47	0.84	0.00
48	0.90	0.00

Table S2. Table of calculated test statistics and *p*-values from the Kolmogorov-Smirnov test applied to each of 48 pairs of an initial array and its inter-array technical replicate produced by Algorithm 1.

Array Number	Statistic	*p*-Value
1	0.06	0.68
2	0.07	0.48
3	0.07	0.54
4	0.12	0.04
5	0.07	0.54
6	0.07	0.41
7	0.05	0.93
8	0.11	0.07
9	0.07	0.48
10	0.09	0.22
11	0.06	0.75
12	0.05	0.88
14	0.04	0.98
15	0.06	0.75
16	0.07	0.48
17	0.04	0.99
18	0.05	0.82
19	0.04	0.96
20	0.07	0.48
21	0.08	0.36
22	0.04	0.96
23	0.04	0.98
24	0.05	0.88
25	0.10	0.12
26	0.05	0.88
27	0.05	0.82
28	0.04	0.98
29	0.06	0.61
30	0.04	0.96
31	0.07	0.54
32	0.04	0.98
33	0.05	0.88
34	0.04	0.98
35	0.07	0.41

Table S2. *Cont.*

Array Number	Statistic	*p*-Value
36	0.06	0.61
37	0.06	0.68
38	0.02	1.00
39	0.05	0.82
40	0.05	0.93
41	0.13	0.02
42	0.05	0.93
43	0.06	0.68
44	0.06	0.75
45	0.05	0.82
46	0.06	0.68
47	0.04	0.98
48	0.05	0.93

Table S3. Table of calculated test statistics and *p*-values from the Kolmogorov-Smirnov test applied to each of 48 pairs of an initial array and its artificially phosphorylated inter-array technical replicate produced by Algorithm 2.

Array Number	Statistic	*p*-Value
1	0.07	0.54
2	0.08	0.31
3	0.12	0.04
4	0.17	0.00
5	0.12	0.03
6	0.10	0.12
7	0.09	0.22
8	0.12	0.04
9	0.12	0.03
10	0.08	0.36
11	0.06	0.75
12	0.09	0.26
13	0.08	0.36
14	0.05	0.82
15	0.08	0.31
16	0.07	0.48
17	0.09	0.26
18	0.10	0.15
19	0.08	0.36
20	0.11	0.07
21	0.10	0.10
22	0.07	0.54
23	0.08	0.31
24	0.08	0.36
25	0.12	0.03
26	0.08	0.36

Table S3. *Cont.*

Array Number	Statistic	*p*-Value
27	0.08	0.31
28	0.07	0.41
29	0.06	0.61
30	0.08	0.31
31	0.08	0.36
32	0.06	0.75
33	0.07	0.41
34	0.07	0.54
35	0.10	0.15
36	0.10	0.15
37	0.09	0.18
38	0.04	0.98
39	0.04	0.98
40	0.09	0.26
41	0.15	0.00
42	0.07	0.48
43	0.10	0.10
44	0.06	0.61
45	0.07	0.54
46	0.07	0.41
47	0.07	0.54
48	0.09	0.22

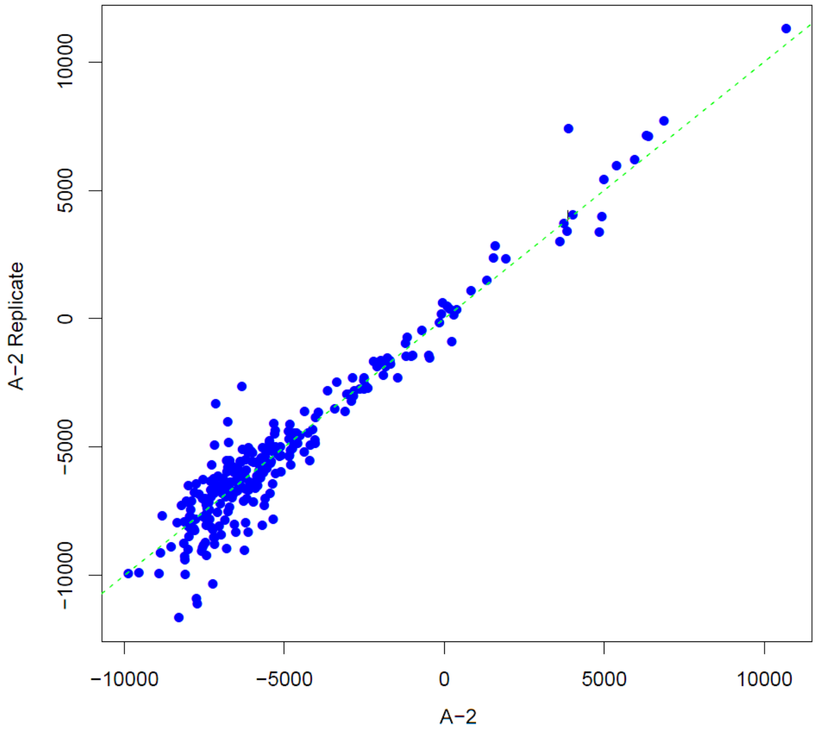

Figure S1. Scatter plot of background-corrected intensity values for an array and its synthesized inter-array replicate produced by Algorithm 1 with $T = 2$ starting with a template array "A-2".

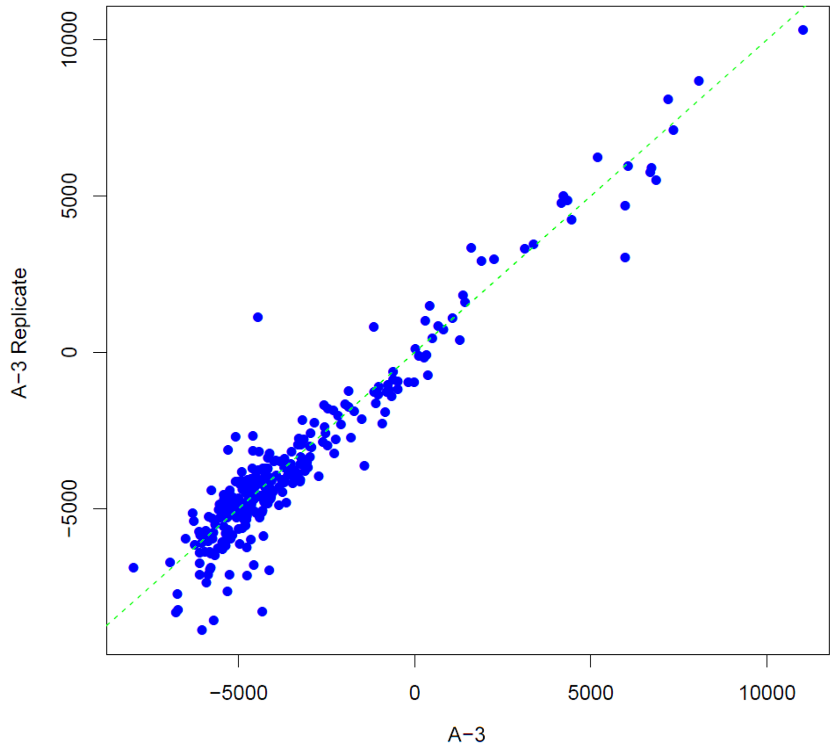

Figure S2. Scatter plot of background-corrected intensity values for an array and its synthesized inter-array replicate produced by Algorithm 1 with $T = 2$ starting with a template array “A-3”.

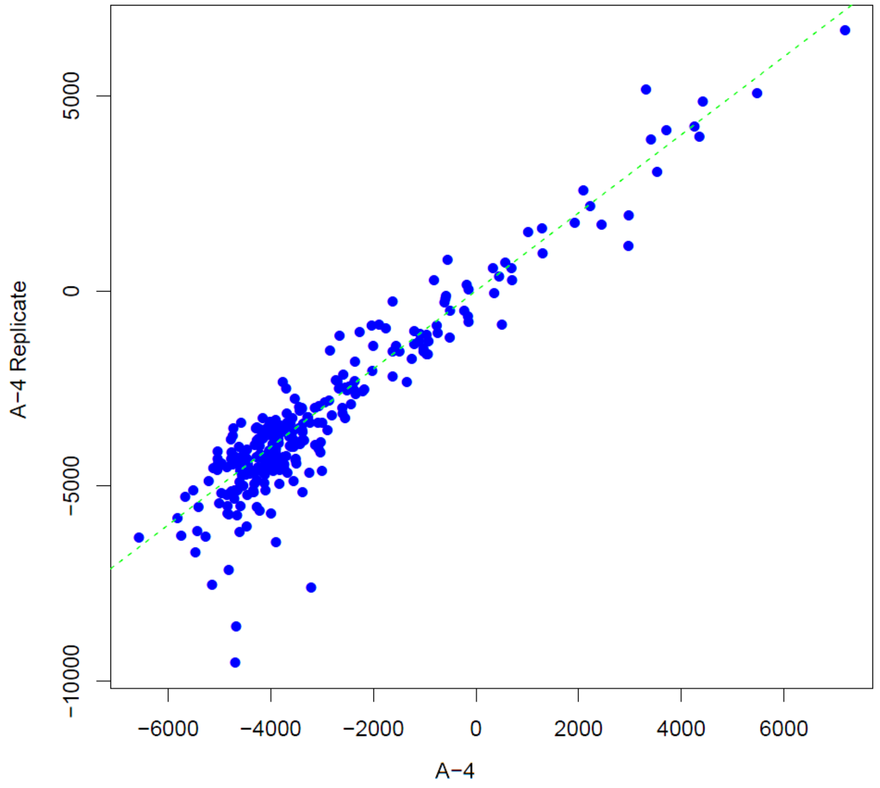

Figure S3. Scatter plot of background-corrected intensity values for an array and its synthesized inter-array replicate produced by Algorithm 1 with $T = 2$ starting with a template array “A-4”.

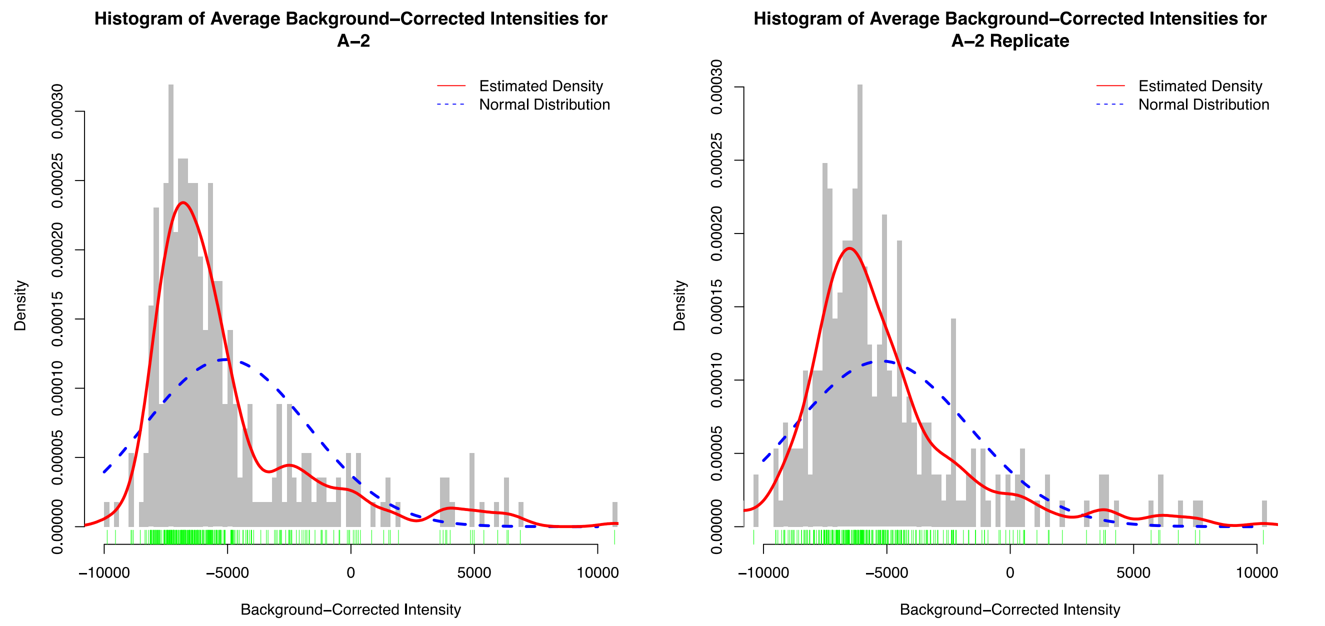

Figure S4. Histogram of background-corrected intensity values for the actual kinome array "A-2" (left), and its inter-array technical replicate (right). The green bars show a one-dimensional plot of background-corrected intensity values.

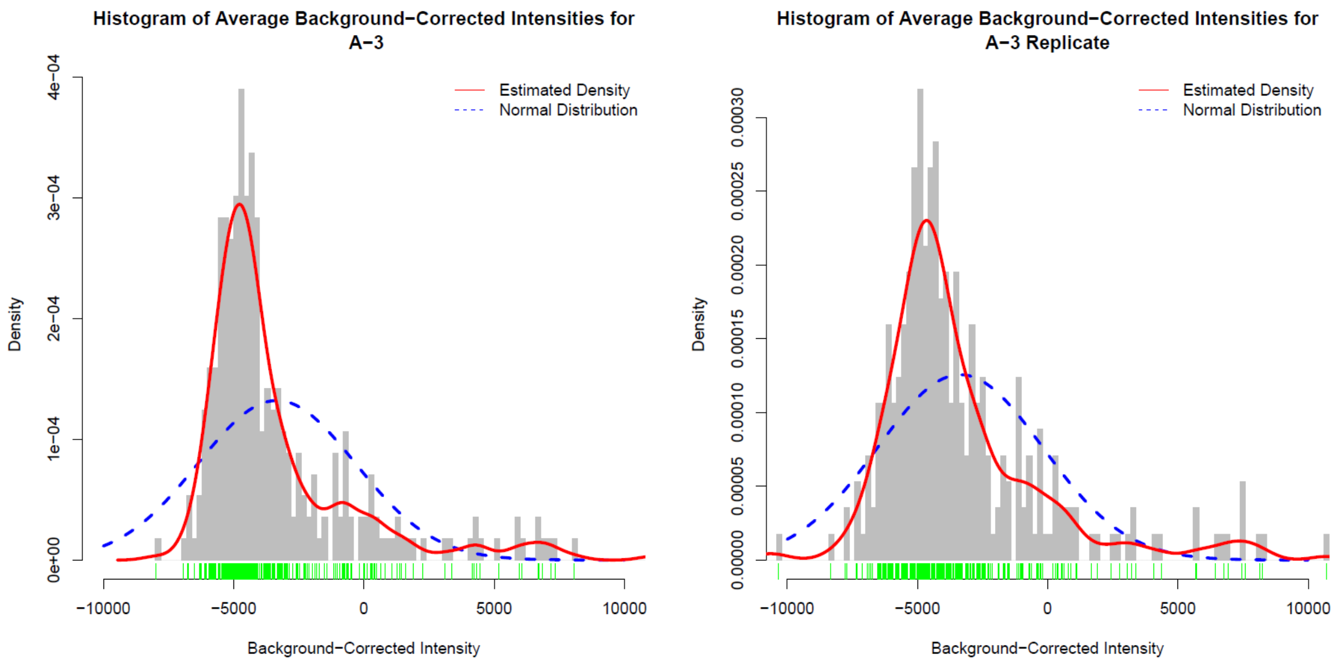

Figure S5. Histogram of background-corrected intensity values for the actual kinome array "A-3" (left), and its inter-array technical replicate (right). The green bars show a one-dimensional plot of background-corrected intensity values.

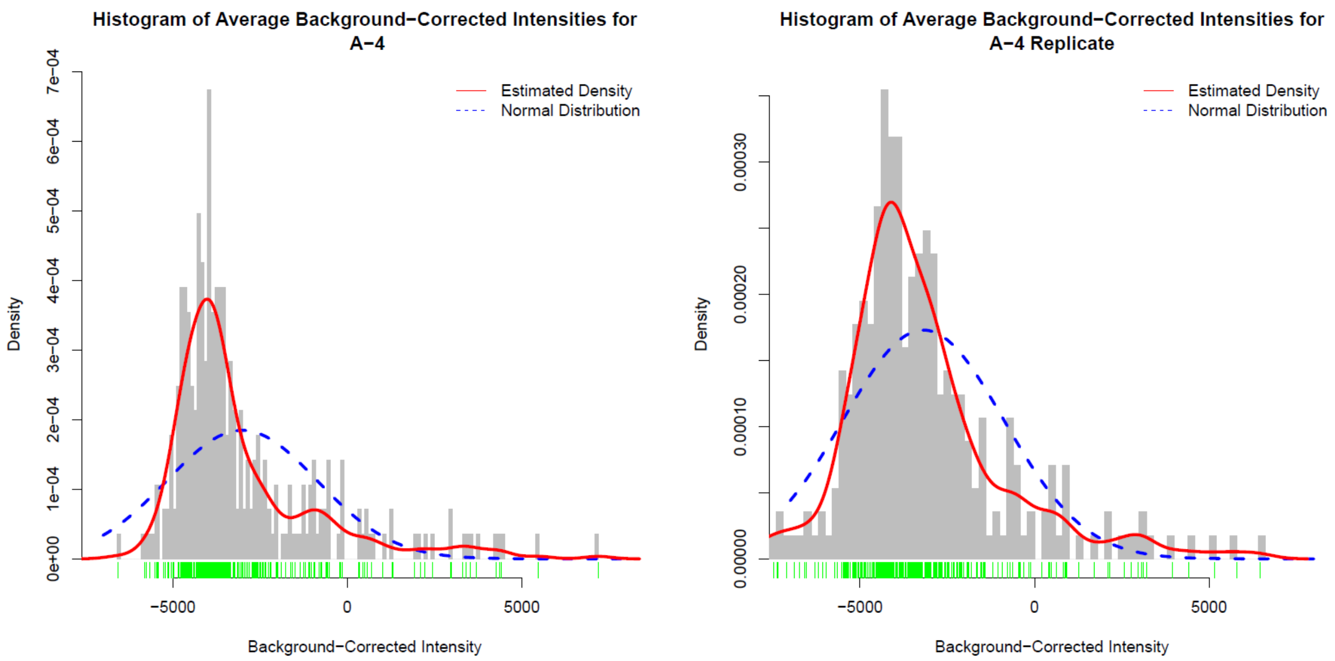

Figure S6. Histogram of background-corrected intensity values for the actual kinome array "A-4" (left), and its inter-array technical replicate (right). The green bars show a one-dimensional plot of background-corrected intensity values.

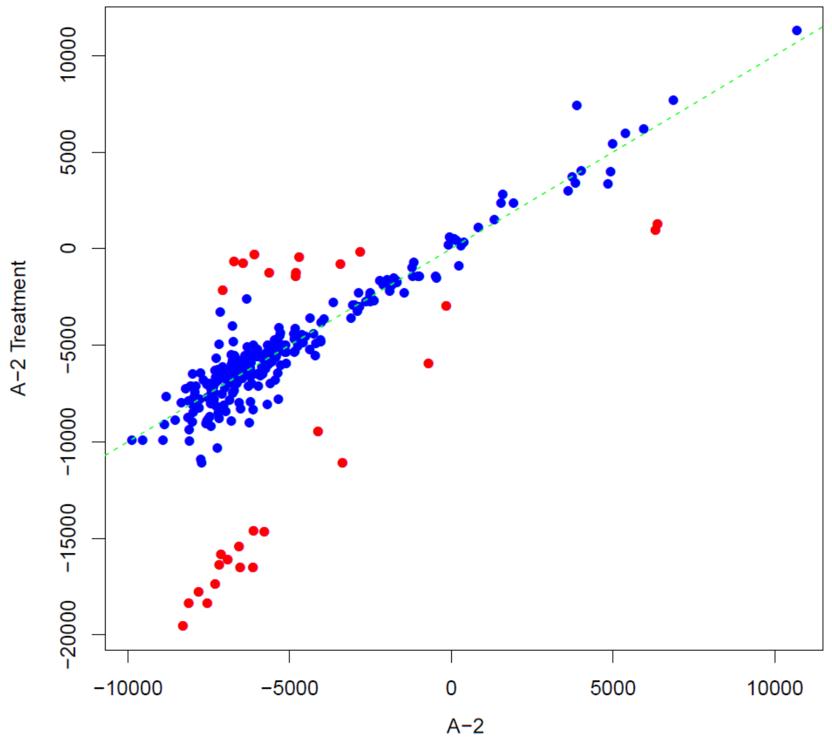

Figure S7. Scatter plot of background-corrected intensity values for an array and a phosphorylated version of its synthesized inter-array replicate when $T = 2$ starting with a template array "A-2". Differentially-phosphorylated peptides are depicted in red.

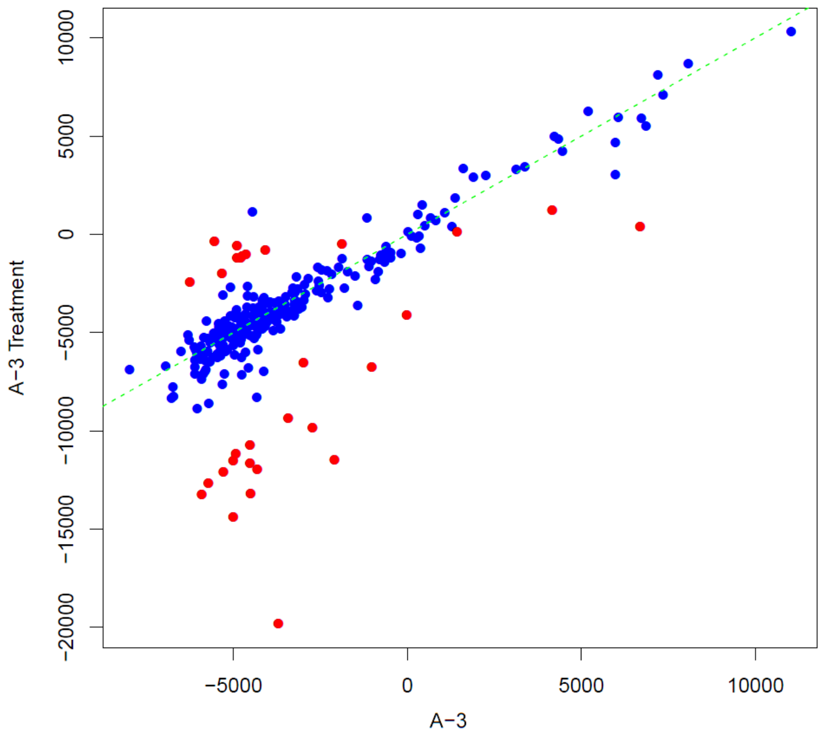

Figure S7. Scatter plot of background-corrected intensity values for an array and a phosphorylated version of its synthesized inter-array replicate when $T = 2$ starting with a template array "A-3". Differentially-phosphorylated peptides are depicted in red.

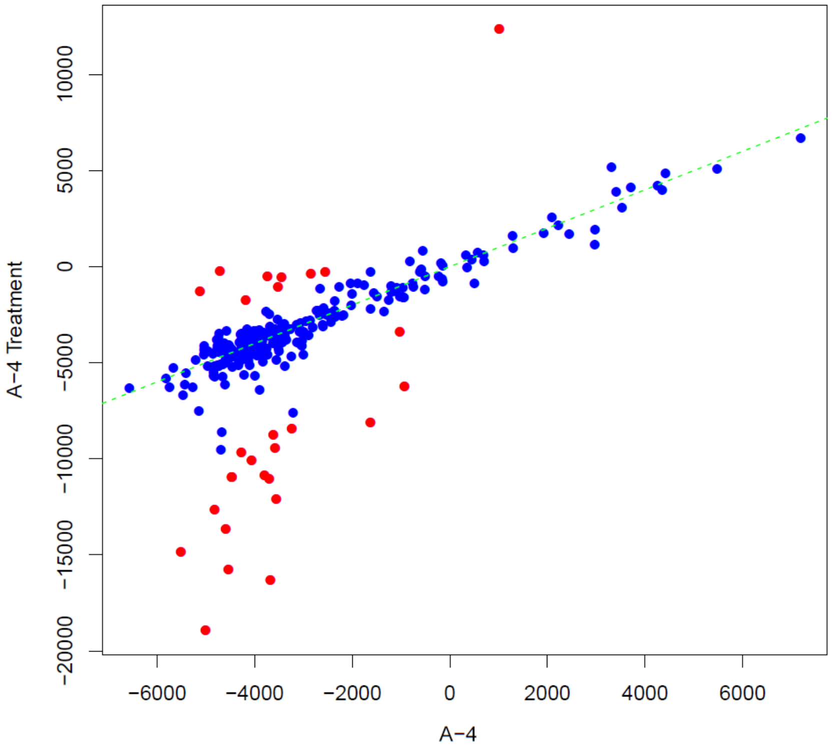

Figure S8. Scatter plot of background-corrected intensity values for an array and a phosphorylated version of its synthesized inter-array replicate when $T = 2$ starting with a template array "A-4". Differentially-phosphorylated peptides are depicted in red.
